# “Backlash!”? A qualitative exploration of hospice palliative care staff’s ongoing experiences of “living with covid”

**DOI:** 10.1177/26323524241283064

**Published:** 2024-09-30

**Authors:** Rebecca Evans, John MacArtney

**Affiliations:** Warwick Medical School, University of Warwick, Coventry, UK; Warwick Medical School, University of Warwick, Medical School Building, Coventry, West Midlands CV4 7AL, UK

**Keywords:** Covid-19, healthcare staff, hospices, qualitative research, sociology

## Abstract

**Background:**

“Living with covid” has meant that the SARS-CoV-2 virus has become a background concern for many in the United Kingdom. However, people with terminal conditions remain some of those at higher risk of Covid-19 affecting the quality-of-life left, as well as the amount of life. Little is known about how staff manage the ongoing risks and challenges—to themselves and those they seek to support—when providing palliative care in the context of an airborne transmissible virus.

**Objective:**

To explore the experiences of UK hospice staff of “living with covid” to identify how Covid-19 continues to affect their work and well-being.

**Design:**

An interpretivist qualitative interview study.

**Methods:**

Reflexive thematic analysis of semi-structured online interviews with 12 staff recruited from three hospices in the West Midlands, UK.

**Results:**

We explored how participants’ accounts of “living with covid” included several ambivalences: Participants not only sought to assert the importance of not forgetting that time but also wished to “move-on.” This included moving Covid-19 to the “background” through embedding systemic adaptions and lessons learnt, while also recognizing that they had to address issues relating to Covid-19 “case-by-case.” Finally, participants’ wish to move-on and a mostly reactive approach to mitigations meant that they were unable to meaningfully reconcile how asymptomatic transmission promotes patients’ quality-of-life left.

**Conclusion:**

Recollections of the difficulties of the Covid-19 public health emergency were part of a “backlash” to any future consideration of mitigations for airborne transmissible viruses and helped justify a “living with (getting) covid” approach. However, this also created uncertainty of how best to support patients who are vulnerable to having the quality and amount of life left compromised by viral infection. The pandemic has brought renewed impetus to re-examine hospice palliative care’s ideals and practices in the context of airborne transmissible viruses.

## Background

During the emergency public health period of the Covid-19 pandemic (March 2020–July 2021) hospice staff, like healthcare staff in other sectors, experienced significant transformation and upheaval to how they worked as part of measures to mitigate infections.^1,2^ Accounts of the emergency period highlighted the difficult and emotional experiences of hospice staff,^3^ as well as the significant effects on patients’ and carers’ holistic, timely care that supported their quality-of-life left.^4,5^ The ending of the public health emergency period led to a transition to guidance that was orientated to “living with Covid-19,” summarized as the “removing domestic restrictions while encouraging safer behaviours through public health advice, in common with long-standing ways of managing most other respiratory illnesses.”^6^ It is in this context that hospices have sought to find the balance between a return to pre-Covid-19 practices and adapting to the ongoing presence of Covid-19.^5^

Hospices are not alone in this. Covid-19 has been a catalyst for improving knowledge about how airborne transmissible diseases can be mitigated by individuals, healthcare organizations, and society.^7^ However, the pandemic has also reiterated the importance of social, political and historical contextualization of why having scientific knowledge about airborne transmissible diseases transmission—including colds, flu, pneumonia, and respiratory syncytial virus—does not necessarily result in appropriate mitigation.^8,9^ Moreover, Covid-19 has refreshed marginalized, but longstanding, concerns regarding public health policy that contains assumptions about which bodies are able to “live with” (repeated) viral infection,^10^ as well as introducing a new intersectional vector that exacerbates pre-pandemic concerns about healthcare inequities.^11^

It is in this context that mitigating airborne viral infections could be understood to be an especially salient and pressing issue for hospice palliative care. Hospice patient populations include higher proportions of people who are potentially highly vulnerable to airborne transmissible diseases.^12^ Moreover, palliative care’s ideal of quality-of-life left is a fundamental part of its identity,^13^ one that the early stages of the pandemic posed an “existential challenge” to.^5^ Yet, since the ending of the emergency period, there has been little critical exploration of the ongoing implications of Covid-19 on hospices or what the collective consequences of the distress and difficulties experienced might entail for any pandemic future.^14–17^ The aim of this study was therefore to explore the experiences of UK hospice staff of “living with covid” to identify how Covid-19 continues to affect their work and well-being.

## Methods

### Design

An interpretivist qualitative interview study best allowed us to explore and contextualize staff experiences and provide an understanding enriched by connecting the micro with the macro.^18,19^ We used a Reflexive Thematic Analysis methodology as this emphasizes the shared meanings to be found in descriptions of experiences and so used Reflexive Thematic Analysis Reporting Guidelines (Braun and Clarke, 2024) to inform our write-up.^20^

### Ethics

Approval was granted by the University of Warwick Biomedical and Scientific Research Ethics Committee (Ref: BSREC 137/22-23) on the 12 October 2023.

### Recruitment

All 13 non-National Health Service (NHS) adult hospices in the West Midlands were emailed inviting them to participate in the study. Inclusion criteria for interview were current staff working at a non-NHS adult hospice in the West Midlands, who were 18-years old or over. A member of hospice staff approached eligible staff either via email or in-person and provided them with a Participant Information Leaflet. We purposively sampled a range of staff roles and sought participants from a diverse demographic sample.

### Data collection

Participants contacted the interviewer (RE) via email to arrange an interview. Demographic information (gender, race, ethnicity, age range, job title and disabilities) was collected prior to the interview to monitor the diversity of the participant sample and as a proxy indicator of seeking a range of experiences. Semi-structured interviews were conducted using MS Teams. Verbal consent was provided on a separate recording at the start of each interview. Interviews used a topic guide that had been developed by drawing on recent literature that explored the factors that affected hospice staff experiences of palliative care provision during the Covid-19 pandemic.^5,21^ Interviews started with an open invitation to tell the interviewer, “What does ‘living with Covid-19’ currently mean for you in your role at the hospice?” The interviewer followed-up with prompts for further information that were appropriate to the participant’s account. For example, questions about balancing infection risks at home and work, how they felt about the emergency period now, and their ongoing experiences with infection control. Digital transcriptions were cross-checked with video recordings of the interview for accuracy, before the video was deleted. The transcriptions were then pseudonymized by removing key identifiers such as colleague and place names, and replaced with participant code numbers and generic name places (e.g. Hospice A).

### Analysis

A Reflexive Thematic Analysis method was used to analyse the data,^22^ as this allowed us to take iterative build-and-revision approach to themes as new interviews were completed. Our analysis addressed the six-steps by first, familiarizing ourselves with the data through checking interview transcripts. This then allowed for the generation of descriptive codes, using QSR International NVivo 1.7.1 to aid the labelling of the data, by finding areas of meaning in the text and organizing the data into “chunks”. From this, candidate themes were then developed. These drafted themes were then revised and defined as more data was collected and interpreted. The researchers—a medical student and sociologist—shared and discussed interpretations throughout the data collection and analysis process, which included returning to the literature during the drafting and editing the paper. This included issues around emotional and psychological effects of working during the first two years of the pandemic, as well as issues relating to how “living with covid” was normalized (or not) and how this was affecting ongoing working practices. As the analysis progressed, we were able to identify several social and normative ambivalences,^23^ that is, the coexistence of opposing attitudes, feelings, discourses, etc. across codes, which we brought together to generate three themes.

## Results

Four hospices responded with an initial expression of interest. Three hospices were able to recruit a total of 12 participants—6, 3 and 3 participants from Hospices A, B and C, respectively. Interviews took place in November and December 2023 and lasted between 30 and 45 min. Participants were drawn from in-patient, day and community services and included one palliative care doctor, seven from nursing services, two Allied Health Professionals, and two non-clinical support staff. Eight staff identified as female and one as non-binary, and all as white. Further breakdown of participant characteristics is provided in Table 1.We report these characteristics in aggregate as detailing each of a participant’s demographic characteristics with their quotes could compromise participant identity (Braun and Clarke, 2024).

**Table 1.** Demographic characteristics for the participants in this study.

Category	Characteristic	Number of participants (%)
Gender	Male	3 (25)
	Female	8 (67)
	Non-Binary	1 (8)
Age range	20 or below	0 (0)
	21–30	2 (17)
	31–40	1 (8)
	41–49	3 (25)
	50+	6 (50)
Ethnicity	White British	11 (92)
	White Irish	1 (8)
Disabilities	None	12 (100)
Religion	None	7 (58)
	Christian	2 (17)
	Christian (other)	2 (17)
	Buddhist	1 (8)
Role	Doctor	1 (8)
	Nursing services	7 (58)
	Allied Health Professionals	2 (17)
	Non-clinical	2 (17)

### Reflexive Thematic Analysis findings

We found asking staff about “living with covid” brought forward many ambivalences, dissonances or contrasting understandings of what it meant for them as private individuals, as staff in the hospice, and in relation to the challenges hospices face when mitigating airborne infectious viruses. For those staff who worked through the emergency public health period their recollections contained multiple assessments of that time. Staff both sought to assert the importance of not forgetting that time, but also wished to “move-on” (P10). This included moving Covid-19 to the “background” (P6) through embedding systemic adaptions and lessons learnt, while also recognizing that they had to address issues relating to covid “on a day-to-day basis” (P3). The largely reactive approach to infection control appeared to be a satisfactory compromise for some. However, other staff struggled to justify their approach to infection control when they reflected upon mitigating asymptomatic transmission, while seeking to fulfilling hospices desire to promote quality-of-life left.

#### Traumatic memories, moral injury, and backlash: The ongoing effects of the Covid-19 emergency period on hospice staff

Participants’ accounts of the emergency public health measures contained considerations of how they viewed that time now, as well as reflections on what it meant for that period to end. As the following participant said about the emergency period,

It hasn’t been forgotten, but for those few years, it’s like been put in the past now and then we can move-on and treat these patients properly like they were treated before covid. (P10)

In this theme we explore a productive tension between how staff seek to acknowledge and speak about the range of difficulties they faced during the emergency period, while also drawing on those experiences to provide a strong justification to “*move-on*.”

When reflecting on the emergency public health period many participants who had worked in hospices during that time used expressive negative terms to describe it, such as, “*impersonal and uncompassionate*” (P11), “*horrendous*” (P4), “*frightening*” (P10), and “*absolutely grim*” (P12). Participants also explained the ongoing emotional effects on themselves and colleagues, “*people still talk about it and, as I got emotional about that, I still do*” (P1). Another participant said, “*But I think when people do talk about it and look back, I think it still effects a lot of the staff, you know, you can see that when they mention it*” (P9). As well as the affective effects another participant described another significant implication of working through the emergency period that they had witnessed, “*I mean, in our team and there is only me left of the original team. Everybody else had left, so it did have a big impact*” (P11).

The following participant exemplified what it was that brought out such a strong reaction to recalling the emergency public health period, “*we felt that we couldn’t do our job properly . . . which then let the patients down. I think that’s the saddest part of it all*” (P11). This is not to say the actions taken during the emergency period held wholly negative memories. Participants also recognized that the guidance and mitigations had benefits. For example, they helped to keep staff “*safe*” but, “*I think we probably went a little bit too far with our caution, but my staff have thanked me for keeping them safe, which is great*” (P1). Importantly, many of the mitigations were seen to compromise what participants saw as key aspects of providing hospice palliative care. As this participant said,

I think we shouldn’t lose sight of that. The human touch that we’re so renowned for, just didn’t happen . . . People couldn’t stay, people couldn’t visit. And the whole ethos of the hospice just disappeared in that in one decision. (P4)

One of the consequences of their experiences was that some participants felt that they or their colleagues might still need emotional and mental health support, “*I guess there’s still issues, aren’t there with the medical profession? I think it’s still trying to recover after everything that’s gone on but, yeah, it’s really hard*” (P8). Another participant explained, “*I felt like, we still had quite a lot of trauma from things, like having to tell visitors that they couldn’t come*” (P5). It was particularly notable that the use of psychological language, such as “*trauma*,” to emphasize the degree of personal and emotional difficulties experienced. One participant reflected on how she had heard a talk about “*moral injury through covid*” and it had resonated with her, “*I think psychologically [the pandemic] has scared a lot of healthcare workers because of what we had to do and enforce*” (P7).

It is against this backdrop of emotional distress and psychological “*trauma*” that evaluations of the emergency period pivoted. The following participant recalled how the emergency period was an intense and difficult time, and how its ending brought feelings of being released from the context that brought such traumatic experiences, as well as hope to return to pre-pandemic ways of living and working.

I think it just boils back to what we’ve all just been through, what we’ve all just had to do, abide by the rules, which I think most of us did, didn’t we. Now it’s like “Woo hoo!,” it’s like it’s all done and dusted, in the past. Let’s just get on with life. (P4)

Another participant reflected, “*. . . there’s a lot of resentment towards the government. . . it just was a horrendous time period and thankfully we’re out of that now*” (P12). That is, there was a strong association in participants’ accounts between the emotional, practical, and clinical difficulties experienced during the emergency period being and a desire to “*just get on*” and put Covid-19 “*in the past*.”

Although there was a desire to “*move-on*,” it was harder to place Covid-19 in the past, as the post-emergency “living with covid” guidance allowed for re-introduction of mitigations. But as the following participant explained, there was some reluctance to re-engage protections such as visitor restrictions, PPE rules, and testing as they did not want to have to face the “*backlash from family and relatives and all*” (P4). Another participant described a broader feeling of returning to any discussion of Covid-19 mitigations saying, “*I think there is a certain covid fatigue, I think probably that exists*” (P2).

The “*fatigue*” of having to address issues relating to Covid-19, the “*resentment*” of having to (unnecessarily) endure that, and a concern about a “*backlash*”—both from patients and carers, but also from hospice colleagues—were contrasted with the hope and desire to return to pre-pandemic ways of working. In what follows, we further explore how recollections of the emergency period affected participants understanding of what moving-on meant while “living with covid.”

#### Normalizing “living with [getting] covid” and being (un)aware of new ways of working

Participants described the ways that their experiences of the emergency period during the pandemic affected how they were “living with covid” at the hospice. Although some participants emphasized how they sought to return to pre-pandemic ways of working, they also described changes to their well-being, attitudes, clinical practice and to the hospice that the pandemic had brought. This is exemplified by the following participant, who described what “living with covid” meant for her,

I understand that to mean that, you know, it’s around us. We know it’s there and we have to live with it and just go through our daily life without sort of thinking too much about it now, because the pandemic is sort of over, but we’ve still got covid around us. It’s about dealing with it on a day-to-day basis as it comes, isn’t it, and how we manage our patients and how we manage ourselves. (P3)

As this participant shows, normalizing “living with covid” entailed managing an important ambivalence: There was both a desire to place Covid-19 in the background of *their* life and “*move-on*” from it being a focus of their work, but there was also a day-to-day recognition that Covid-19 could potentially have significant effects upon them and the hospice’s patients.

Participants described how “living with covid” meant finding ways to put it out of their mind or minimize its meaning for them as just “*part and parcel of life*” (P2). For the following participant, this included a recognition that living with Covid-19 meant living with *getting* covid.

I mean, we’re all still at risk of it. It’s just one of those things now, isn’t it? That we just have to live with. . . if I’m gonna get it, I’m gonna get it and that’s it. (P11)

An important part of carrying on as normal was the idea that Covid-19 had undergone a change in how participants understood it as posing a risk to them. Some participants highlighted the importance of the vaccine and the reduced numbers of deaths, which helped transform Covid-19 from a “*scary*” (P9) disease, to being likened to existing viruses such as colds or flu.

It was just so, sort of, matter of fact that, it was like, well, okay, well, this is going to be just like another cold, really. It’s just another flu. It just kind of felt obvious when they said it. (P6)

However, when it came to the issues that Covid-19 posed for patients, some participants were more circumspect. As the following participant notes, it was not always as simple as forgetting Covid-19 exists,

And yeah, I’ve probably slipped straight back into normality and kind of forget covid exists. Which is nice. And then, yeah, I’m saying it like that, actually, should I really be doing that? (P12)

Participants described how a legacy of the emergency period was that they were “*more aware now of the risk*” (P2) that Covid-19 and other airborne transmissible diseases posed to others, including patients.

You’re a bit more conscious of going into patient rooms. Just stuff we probably should have been doing anyway, but just a bit more heightened of making sure that, you know, we’re wearing the correct PPE [Personal Protective Equipment] and going into the rooms and things like that. (P6)

However, as we have already seen, that awareness was in tension with the desire to put Covid-19 in the background of life and move-on, as the following participant expressed,

I am aware of it. I don’t talk about it because I think we’re all fed up of talking about it, but I am more mindful of what other people feel and think when I’m mixing really. (P10)

It is important to note that part of what helped some participants “*move-on*” and even “*forget*” Covid-19 was the institutionalization or routinization of many mitigations. Participants noted changes such as the greater use of video communication, better awareness of PPE use, and the ongoing monitoring national guidance and updates of regional and organizational prevalence rates. Similarly, just as individuals sought to forget there was also an institutional desire to “*move-on*” from Covid-19. As the following participant explained,

And it’s really interesting that nobody really is allowed, it’s not you’ve not been told it’s not allowed, but you don’t put anything in about covid in any reports, you know, in our quality account it was always “post pandemic.” Well in the quality count I’ve recently written, we don’t talk about the pandemic because it’s gone, it’s gone now and we’re moving to a different place. (P1)

Living with getting Covid-19 therefore resulted in an ongoing ambivalence of individual and collective (un)awareness, while also being an approach that allowed participants to call upon their experiences and react when needed. As this participant summarized,

[covid] is kind of not really thinking about it until something happens, that I’d maybe think about [doing something]. (P4)

However, as we will explore in the final theme, this reactive approach did not always best accord with the hospice palliative care ideal to promote quality-of-life left.

#### The problem of quality-of-life left when “living with covid.”

In this theme we explore how participants struggled with the ambivalence, juxtaposition—sometimes dissonance—of a “living with (getting) covid” approach and knowing patients were particularly vulnerable to Covid-19 affecting their amount or quality-of-life left.

As the following participant explained, many patients who receive hospice care are particularly vulnerable to Covid-19, or any airborne transmissible virus, detrimentally affecting the quality of their life left,

The greatest risk in the hospice is to those vulnerable patients that we have because obviously they have multiple things that they’re dealing with and to get covid, well to get anything even just a really bad cold, could make their situation a lot worse. So for our patients, they are vulnerable, they are at high risk and that’s why we do monitor things very, very carefully, but we’re not going overboard. (P1)

Other participants described how they had witnessed patients who had been infected with Covid-19 in addition to their terminal illness and how “*it wasn’t a nice end of life*” (P7) and how “*there was quite a lot of suffering*” (P5).

But not all staff accepted that dying with Covid-19 was necessarily problematic. They also drew on experiences of caring for patients dying with airborne transmissible viruses, such as patients with Chronic Obstructive Pulmonary Disease, who can die of a (untreated) pneumonia infection,

So, it’s difficult, you know, why are we making more now of somebody contracting covid and then they die, but, actually, they were very weak and vulnerable anyway and they would have died, or they could have died if they contracted something like pneumonia? (P3)

Another participant reflected,

It sounds bad, but lot of these patients are gonna die in the next couple of weeks anyway. But if they had covid in and that happened, if we isolated them and stopped them seeing their relatives in their final two weeks of life, that would just be horrendous for everyone involved. That actually, even if they die, catch covid and die a little bit quicker, for some of them [patients], that’s an acceptable risk. (P12)

What is important is that, even within this position, we can see how an interest in the participant’s quality of life—expressed through a concern to see relatives—is a key frame though which to assess what Covid-19 mitigations are acceptable or not.

As well as bringing attention to how hospice palliative care can be understood to be promoting quality-of-life left in the context of airborne transmissible disease, the pandemic has also increased awareness of the difficulties of sharing responsibility for those decisions with patients, especially in the context of an asymptomatic disease. Like the previous participant, mitigating Covid-19 transmission was often equated with maintaining the emergency period guidance, which was seen to be unacceptable given its effects upon patients’ quality of life. Other participants recognized that some mitigations were necessary, but these would be applied on “*case-by-case*” (P7). The following participant explained that “*case-by-case*” meant “*if people need [mitigations], then they will receive it*” (P9) and that “*you make all those decisions with patients*” (P12). Covid-19 mitigation was therefore predicated on staff and patient’s knowing and sharing their infection status and the patient’s preferences for risk and understanding of what “living with (getting) covid” might mean for them. Should Covid-19 be suspected, and this was held to be problematic for either party, some form of mitigative action could then be taken.

However, when we asked participants about asymptomatic transmission, the tensions we have explored in our analysis so far again resurfaced. The wish to “*move-on*” in a state of (un)awareness meant that when the following participant was asked about wearing PPE to prevent asymptomatic transmission she said, “*Well, it depends how you were feeling [symptomatically]*” (P11). Another member of staff was more circumspect about the reactive mitigation status-quo saying, “*That’s something you can’t really avoid.*” She exemplified the current approach to “living with covid” in hospice palliative care when she said,

I think you’ve just got to get on with it. Obviously if you have got symptoms then it’s only right that you take a test and make sure. But I think if you’re asymptomatic, I don’t think it shouldn’t prevent you from carrying on as normal. (P10)

The Covid-19 emergency public health period is over, but participants struggled to reconcile how taking a reactive “living with (getting) covid” approach enabled hospice palliative care to meaningfully promote quality-of-life left in the presence of asymptomatic transmission of an airborne virus.

## Discussion

We have identified several important contributions to understanding how UK hospice palliative care staff’s experiences of “living with covid” continued to affect their work and well-being. First, participant’s recollections of the first two years of the pandemic contained multiple evaluations—emotional, psychological, practical, policy, political, and moral—of the difficulties they faced. Second, while participants described their ongoing need for emotional and psychological support, they also drew on their recollections to provide what they felt were strong and compelling reasons to “move-on” from Covid-19. This included embedding and routinizing some of the changes brought by the pandemic, as part of a reactive “living with (getting) covid” approach. Although, thirdly, participants also recognized how this approach could be inconsistent with the hospice ideal of promoting quality-of-life left, doing more was felt by some to be too much of a return to those ways of working during the emergency period—a time framed by multiple practical, clinical and personal difficulties^1,21^ including, but not limited to, psychological trauma, moral distress and injury.^14–17^ It is in this context that further claims of how dying with a Covid-19 infection should accepted, like flu and pneumonia, as a natural part of the dying process should be examined.

### Returning to the problem of quality-of-life left for hospice palliative care: Implications for practice and future research

The literature exploring the effects of the Covid-19 pandemic emergency period on hospices is limited by the circumstances of its construction: mostly using opportunity samples (staff), insight was often compromised by the need for rapid results leading to an over-reliance upon surveys,^21^ and flat analysis that eschewed the effects of the socio-political context of the pandemic. Research seeking to “learn lessons of the pandemic” will need to pay critical attention to what is at stake for hospice patients in any push to “live with (getting) covid.”^24^ Further analysis will also need to better evaluate the consequences of enforced normalcy,^25^ as well as what compromises this may incur—or bring to the surface—for those seeking to defend hospice palliative care’s ideals and assert the “human rights” of people with life-limiting illnesses.^26^

Given the limitations of the existing hospice palliative care literature and the empirical concerns we have raised about how hospices might be “living with covid,” questions remain about what quality-of-life left now means in relation to airborne transmissible viruses. Our findings suggest that future research may wish to attend to three questions: First, what is the lesson being learnt by the naturalization of people with life-limiting illnesses dying from Covid-19? The pandemic has cast new light on long-standing discussions about medicine’s role when infections hasten death that were largely a question for clinical ethics.^27^ But we found that hospice palliative care’s timely (earlier) intervention in end-of-life care transformed both the location (from deathbed, to day and community settings) and temporality (not hours or days, but weeks, months or years of life left) of these dilemmas. This meant seeing hospice services as vectors of transmission brought feelings of uncertainty, ambiguity and emotional concern for hospice staff. This was particularly evident in conversations about asymptomatic transmission and the current, mostly reactive, mitigation practices.

Second, further attention needs to be given to how responsibility for quality-of-life left is shared in the context of a pandemic virus? That is, what lessons are being learnt through the implementation of practices that prioritize individual risks and responsibilities, in a service that defines itself through a concern for holistic approach to care? In particular, what use is a “case-by-case” approach that is dependent on openness and honesty about infection status, when along with the emotional and moral pull to minimize mitigations that staff described, there are social and economic drivers that push people to hide their symptoms.^28^ Third, if a “living with (getting) covid” approach takes the able and well body as its starting point,^10,24^ how might a “living with not getting covid” approach be implemented by hospices? This is particularly important as hospice palliative care seeks to reduce inequities of access, but now does so in a context where Covid-19 can exacerbate pre-existing disparities.^11^

### Strengths and limitations

Our findings draw on the experiences of 12 hospice staff from one region of the United Kingdom. As an interpretative qualitative study this was sufficient for us to provide in-depth exploration of participants’ experiences.^18^ In doing so, we successfully identified interpretations of what “living with covid” meant for staff at the time of the interview^29^ and demonstrated how these examples can disrupt dominant social positions or normative discourses.^30^ Our analysis has provided a meaningful response to our research aim, along with transferrable insights by re-posing questions about what quality-of-life left means for hospice palliative care.^18^

While we sought a diversity of staff demographics, we were unable to recruit an ethnically diverse sample. The Covid-19 pandemic is known to have a disproportionate effect upon healthcare staff from ethnic minorities, particularly during the emergency period.^11,31,32^ However, further research is needed to explore how the issues we identified might affect hospice staff or intersect with other aspects of staff identity and experiences.^33^

## Conclusion

Many societies around the world are seeking to learn to live with Covid-19 and the (implied) acceptance of getting infected. If hospice palliative care staff are also going to adopt a “living with getting covid” approach then we found that rather than allow staff to “move-on” and “forget” the pandemic, Covid-19 becomes part of day-to-day working and a source of uncertainty and ambivalence about what the right thing is to do (and for whom). We found the wish to avoid further distress associated with mitigations of the pandemic emergency has become entwined with a largely reactive discourse around infection control. This was especially problematic in the context of concerns about asymptomatic transmission of a virus that can still significantly affect the quality and quantity of life left of people with life-limiting conditions.

Four years after the start of the pandemic, there is now much more knowledge about Covid-19 and how to mitigate it, which has implications for how other airborne transmissible viruses could be managed.^34^ This knowledge changes the terms by which actions, such as “living with getting covid,” might be evaluated.^35^ If hospice palliative care is to continue to promote the gold standard end-of-life care, including timely provision of holistic care to promote the quality-of-life left,^13^ it will need to critically and systemically engage with questions of how best to live with not transmitting or contracting airborne transmissible viruses.

## Declarations

**Ethics approval and consent to participate:** Approval was granted by the University of Warwick Biomedical and Scientific Research Ethics Committee (Ref: BSREC 137/22-23) on the 12 October 2023. Vernal consent was provided by participants.

**Consent for publication:** Participants provided consent to be quoted in publications.

**Availability of data and materials:** Interviews contain personally identifiable information. To access please contact the corresponding author.
